# Comment on ‘Effect of Skeletal Muscle Mass and Its Associated Mediators on the Development of Steatotic Liver Disease: A Cohort Study in China’ by Liang et al.

**DOI:** 10.1002/jcsm.70288

**Published:** 2026-04-16

**Authors:** Atsushi Nakamura

**Affiliations:** ^1^ Nippon Koukan Hospital Gastroenterological Liver Disease Center Kawasaki‐shi Kanagawa Japan

To the Editor,

1

We read with great interest the study by Liang et al. examining skeletal muscle mass indices and MASLD development [1]. Their comprehensive analyses are impressive, yet the interpretation of skeletal muscle indices may warrant reconsideration.

## 2 The Data Suggest Adiposity, Not Muscle Mass Per Se, Drives MASLD Risk

Table 1 of the original study reveals a notable pattern: MASLD patients have virtually identical absolute muscle mass (ASM/height^2^: 7.11 vs. 7.07 kg/m^2^) but substantially higher visceral adipose tissue (VAT = 84.6 vs. 114.4 cm^2^, +35%). The reversal of ASM/height^2^ from risk‐increasing (RR 1.67) to risk‐decreasing (RR 0.77) after BMI adjustment likely reflects the removal of adiposity confounding rather than revealing a true muscle effect.

**TABLE 1:** Key discrepancies and recommended analytical adjustments.

Analysis component	Current study findings	Interpretation	Proposed approach
*Baseline comparison* (Table 1) [1]			
ASM/height^2^ (kg/m^2^)	Non‐SLD: 7.11 MASLD: 7.07 (−0.6%)	No difference in absolute muscle mass	Indicates confounding by body size
VAT (cm^2^)	Non‐SLD: 84.6 MASLD: 114.4 (+35%)	Largest between‐group difference	Primary driver of MASLD risk [2]
ASM/VAT ratio*	Non‐SLD: 0.084 MASLD: 0.062 (−26%)	Sarcopenic obesity in MASLD [3, 4]	Use as primary exposure
*Risk prediction* (Table S13) [1]			
VAT (per SD)	Not examined	Strongest predictor	Include in all models [5, 6]
BMI (per SD)	RR 1.51 (1.29–1.77)	Weaker than VAT	Secondary to VAT
HOMA‐IR (per SD)	RR 1.44 (1.29–1.61)	Weaker than VAT	Likely VAT‐mediated [5]
*Association analysis* (Table 3) [1]			
ASM/height^2^ unadjusted	RR 1.67 (1.51–1.84)	Positive association	Reflects body size/adiposity
ASM/height^2^ BMI‐adjusted	RR 0.77 (0.67–0.89)	Association reverses	Adiposity confounding removed
ASM/height^2^ VAT‐adjusted	Not examined	—	Expected: RR ≈ 1.0 (no effect expected) [2, 6]
ASM/VAT ratio*	Not examined	—	Expected: RR reduced to approximately 0.4–0.5 per SD increase [3, 4, 7]

Abbreviations: ASM, appendicular skeletal muscle mass; BMI, body mass index; CI, confidence interval; HOMA‐IR, homeostasis model assessment of insulin resistance; MASLD, metabolic dysfunction–associated steatotic liver disease; RR, risk ratio; SD, standard deviation; SLD, steatotic liver disease; TG, triglyceride; VAT, visceral adipose tissue.

* ASM/VAT ratio calculated as (ASM/height^2^) ÷ VAT × 100 for scaling.

## 3 VAT: The Strongest Predictor, yet Excluded From Primary Analyses

Table 1 of Liang et al.'s study shows that MASLD cases have substantially higher visceral fat area (VFA; 114.4 vs. 84.6 cm^2^, +35%), representing the largest between‐group difference among body composition indices. Supplementary Figure S4 of the same study further demonstrates that higher VFA quartiles consistently confer greater MASLD risk across models [2]. By contrast, Table S13 provides detailed per‐standard‐deviation risk estimates for multiple metabolic markers (e.g., BMI, HOMA‐IR, triglycerides) but not for VFA, preventing direct quantification of its comparative predictive strength. Moreover, VFA was not included as a covariate in any multivariable model evaluating skeletal muscle indices (Tables 3, S6, S9–S11 and S15), making it impossible to determine whether the reported muscle effects are independent of visceral adiposity.

## 4 A simpler Explanation: Sarcopenic Obesity

The data are consistent with sarcopenic obesity—low muscle mass relative to adiposity. From the reported data, MASLD patients show a 26% lower ASM/VAT ratio (0.062 vs. 0.084), indicating insufficient muscle mass relative to their visceral adiposity burden. This single metric captures the essence of metabolic risk better than examining muscle or fat in isolation [3, 4].

## 5 Recommendations for Future Research

We respectfully suggest that future studies—and reviewers evaluating them—should consider the following:

- Include VAT as a covariate in multivariable models examining muscle–MASLD associations to determine whether muscle effects are independent of visceral adiposity.
- Examine the ASM/VAT ratio as a primary exposure, which directly quantifies the muscle‐to‐visceral‐fat balance relevant to metabolic health [3, 4].
- Test VAT as a mediator in the muscle–MASLD pathway, as it may be the primary mechanism linking body composition to liver disease.
- Clarify the biological pathway by incorporating muscle‐derived factors (e.g., irisin and interleukin‐6) [8] rather than relying solely on adipose‐derived markers such as adiponectin [9].

## 6 Conclusion

The authors' data strongly suggest that visceral adiposity is the primary driver of MASLD risk. The apparent associations with skeletal muscle mass indices likely reflect varying degrees of adiposity confounding rather than direct muscle–liver effects. Including VAT in future analyses would clarify whether skeletal muscle mass has protective effects independent of its correlation with visceral adiposity. Until VAT is incorporated into analytic models, the most parsimonious interpretation remains that the muscle‐to‐visceral‐fat ratio—rather than muscle mass per se—defines metabolic risk in MASLD. This perspective does not diminish the clinical importance of maintaining muscle mass but rather emphasizes that interventions should target both muscle preservation and visceral fat reduction simultaneously [10].

Importantly, this perspective aligns with recent MASLD clinical trials [11], in which GLP‐1–based therapies markedly improve liver outcomes despite modest reductions in lean mass—reinforcing that it is the muscle‐to‐visceral‐fat balance, rather than absolute muscle mass, that determines metabolic risk.

## Funding

The author has nothing to report.

## Conflicts of Interest

The author declares no conflicts of interest.
